# Supplementary material for: The structure and function of the global citrus rhizosphere microbiome
Source: Nat Commun. 2018 Nov 20;9:4894. doi: 10.1038/s41467-018-07343-2 (PMC6244077; doi:10.1038/s41467-018-07343-2)
Supplement: Supplementary file 1 — Supplementary Information [file 41467_2018_7343_MOESM1_ESM.pdf]

Supplementary Information

The structure and function of the global citrus rhizosphere microbiome

Xu *et al.*

List of Content

Supplementary Figures 1-14

Supplementary Tables 1-5

Supplementary Figures

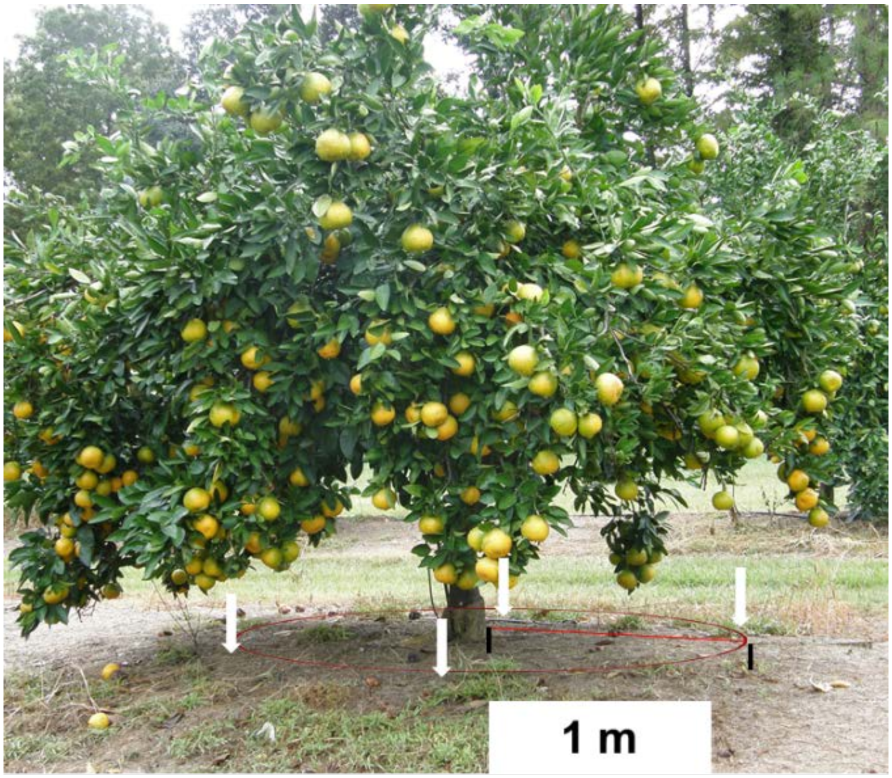

Supplementary Figure 1. The sample collection approach used for collecting citrus rhizosphere microbes. The samples were collected from four corners around 1 meter away from the trunk.

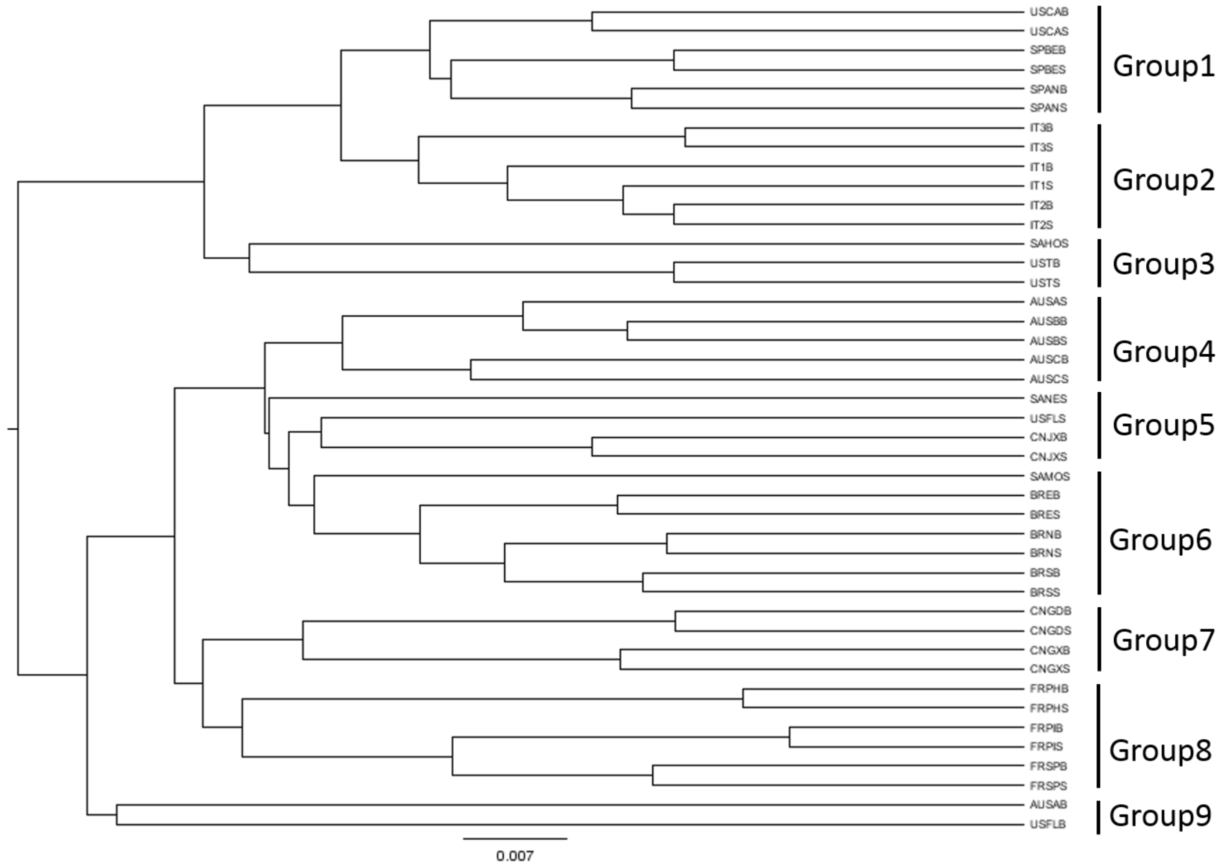

Supplementary Figure 2. The microbial community similarity between samples estimated by Mash based on the metagenomic data.

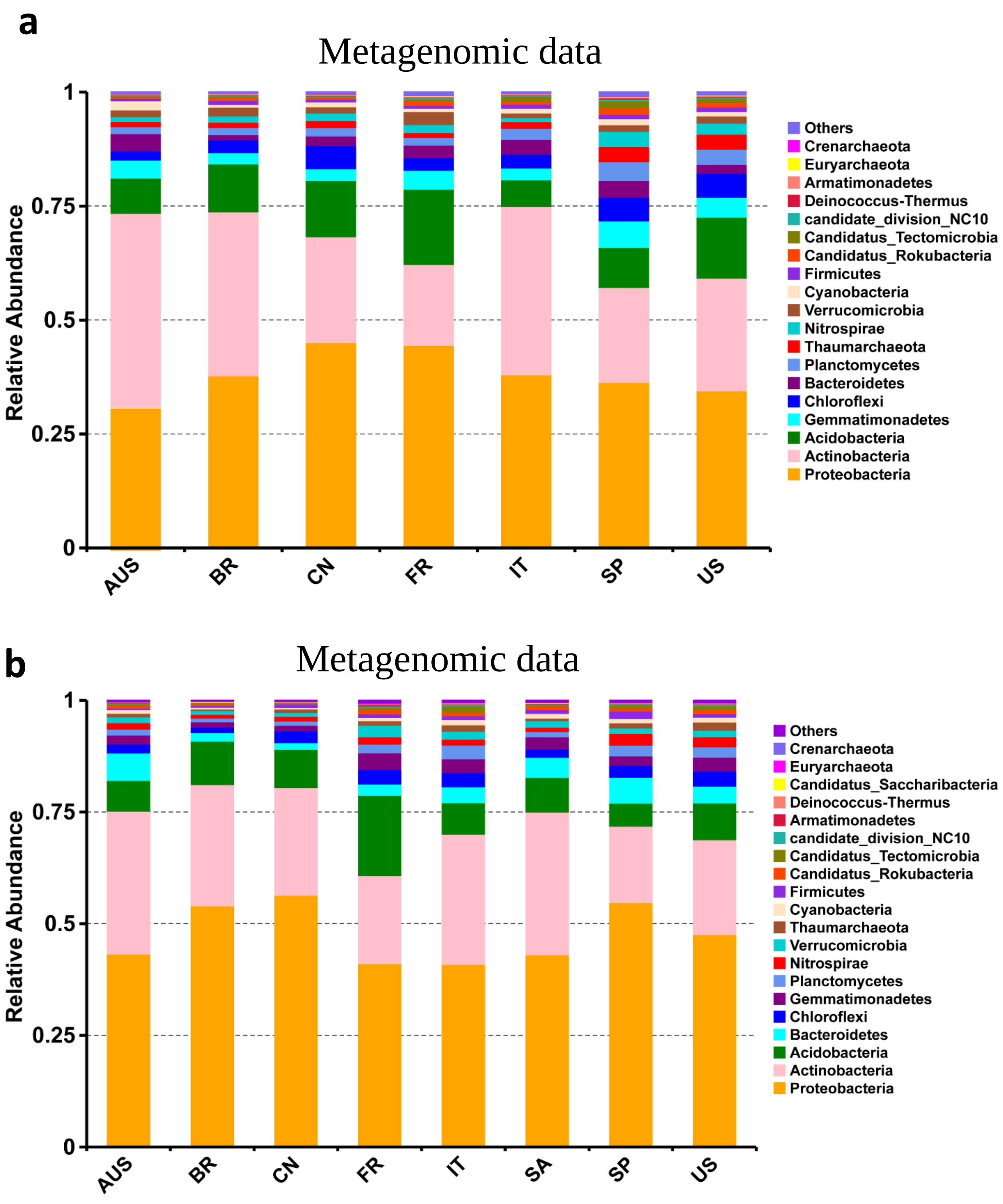

Supplementary Figure 3. Taxonomic distributions of citrus-associated bulk soil and rhizosphere microbiomes across locations at prokaryotic phylum level. (a) The distribution of bacterial and archaea at phylum level for bulk soil samples based on metagenomic data; (b) The distribution of bacterial and archaea at phylum level for rhizosphere samples based on metagenomic data. CN, China; SA, South Africa; BR, Brazil; SP, Spain; FR, French Réunion island; IT, Italy; AUS, Australia.

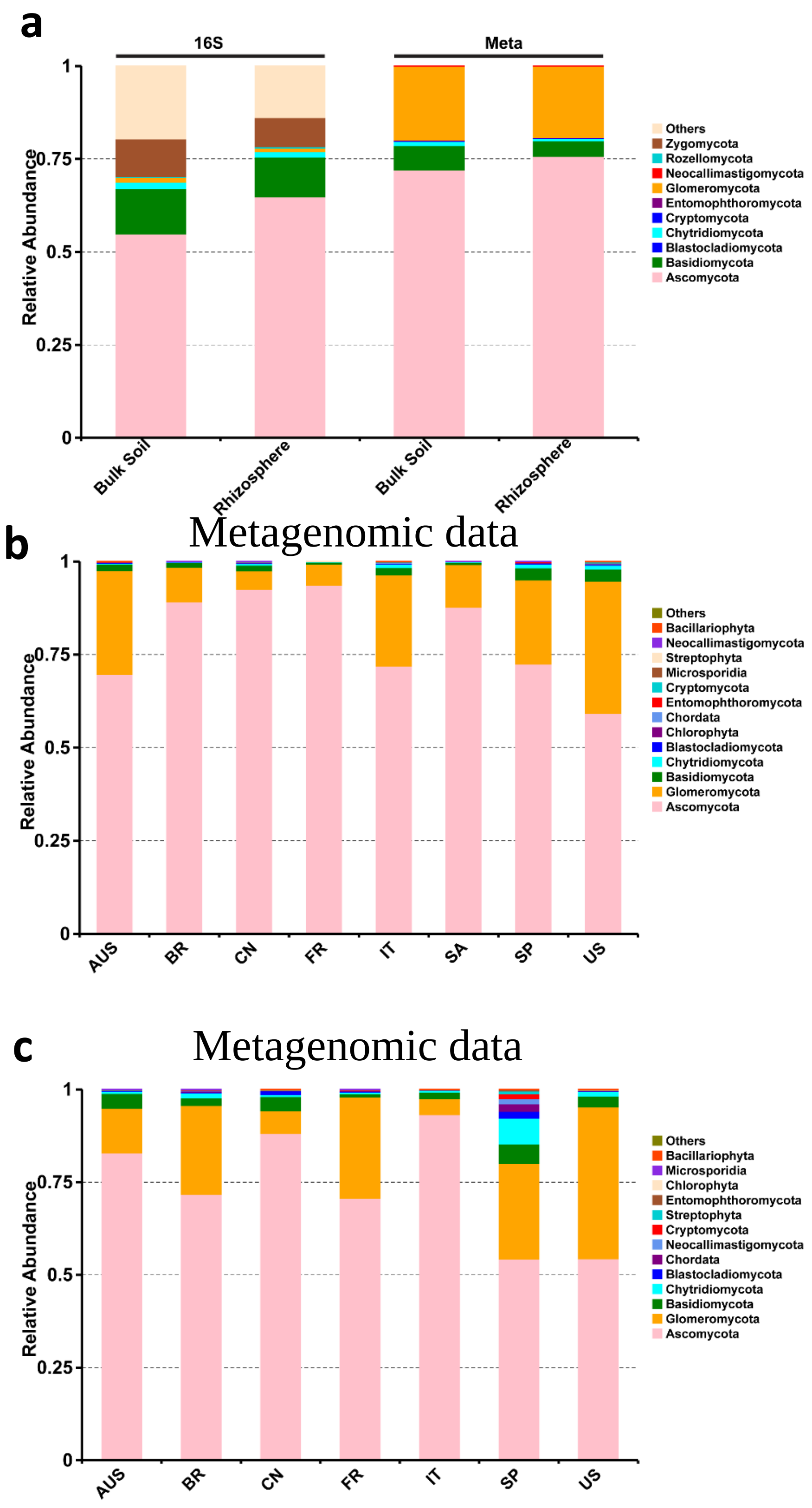

Supplementary Figure 4. Taxonomic distributions of citrus rhizosphere and bulk soil microbiomes across locations at eukaryotic phylum level. (a) Eukaryotic phylum distribution

between bulk soil and rhizosphere samples based on ITS2 and metagenomic data; (b) The distribution of eukaryotic microbes at phylum level for bulk soil samples based on metagenomic data; (c) The distribution of eukaryotic microbes at phylum level for rhizosphere samples based on metagenomic data. CN, China; SA, South Africa; BR, Brazil; SP, Spain; FR, French Réunion island; IT, Italy; AUS, Australia.

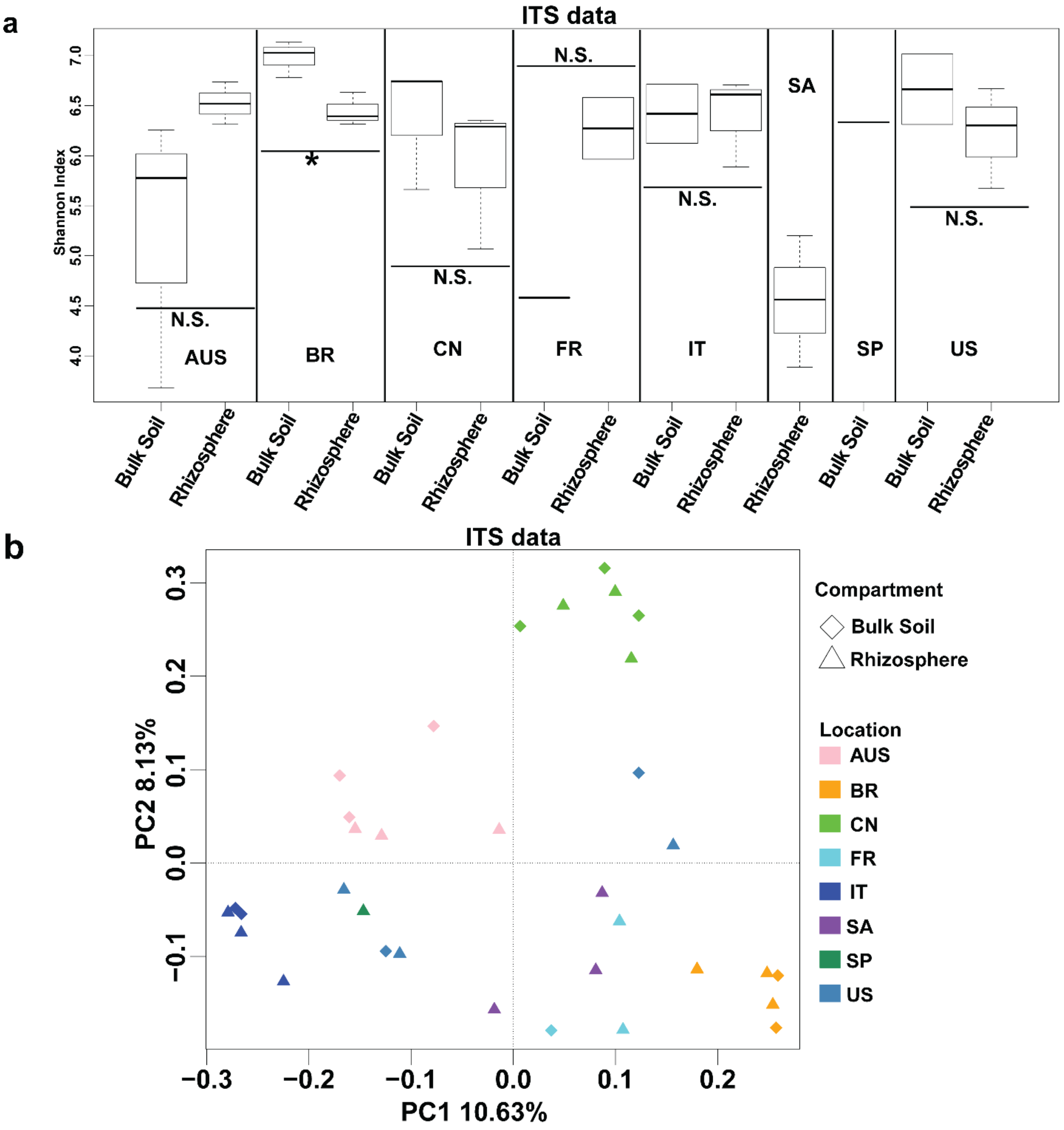

Supplementary Figure 5. Alpha and beta diversity comparisons of citrus rhizosphere and bulk soil microbiomes across compartments based on ITS2 data. (a) Alpha diversity comparison among bulk soil and rhizosphere for each location based on Shannon index; N.S., No significant, *, P-value <0.05, one-sided t-test; center value represents the median of Shannon index; (b) PCoA based on the unweighted unifrac distance among bulk soil and rhizosphere samples for each location. CN, China; SA, South Africa; BR, Brazil; SP, Spain; FR, French Réunion island; IT, Italy; AUS, Australia.

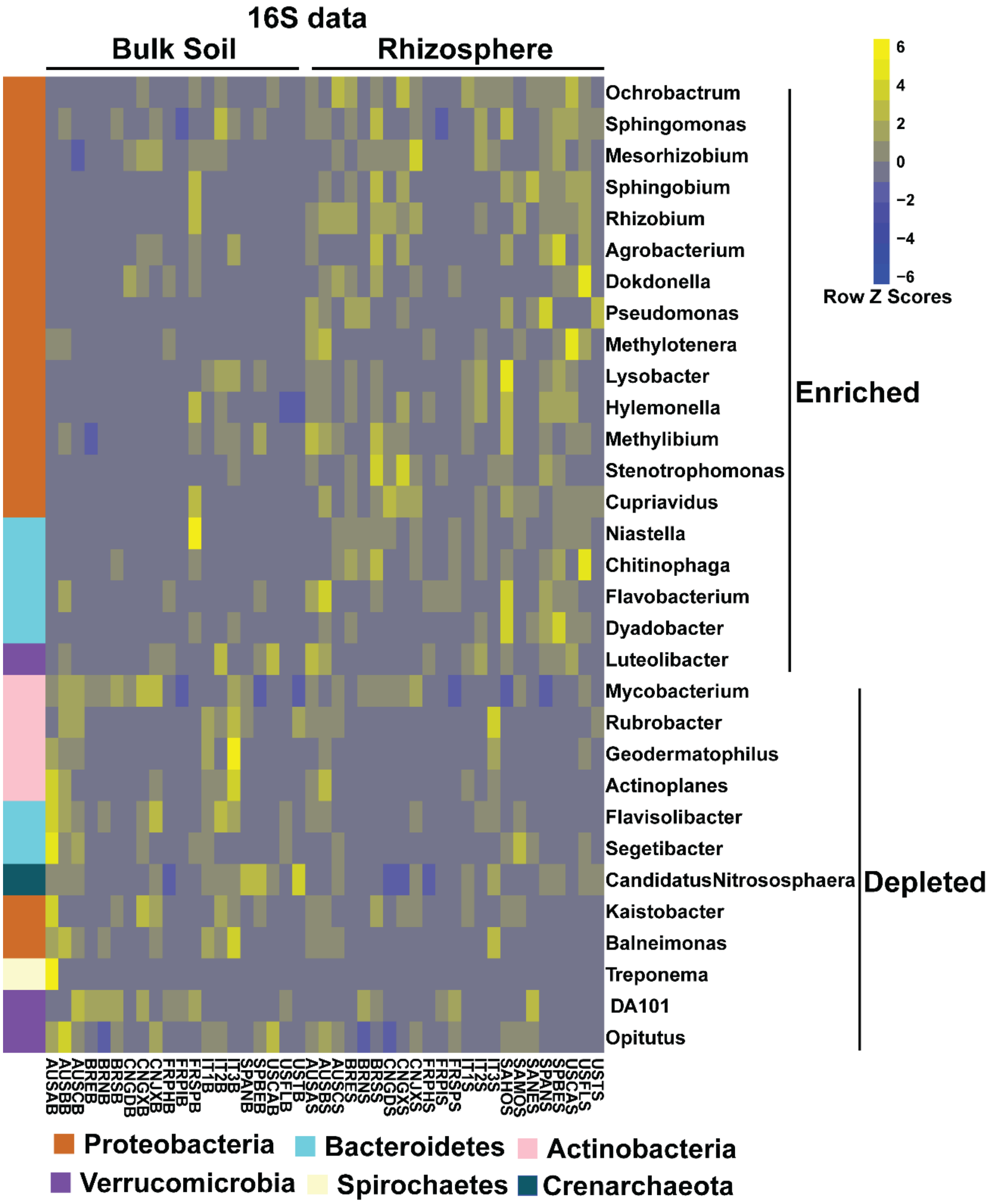

Supplementary Figure 6. The rhizosphere-enriched and rhizosphere-depleted species at the genus level based on 16S data. Scale, relative abundance of genus at row normalization by removing the mean (centering) and dividing by the standard deviation (scaling). The color from blue to yellow represents a relative abundance of each taxa from low to high.

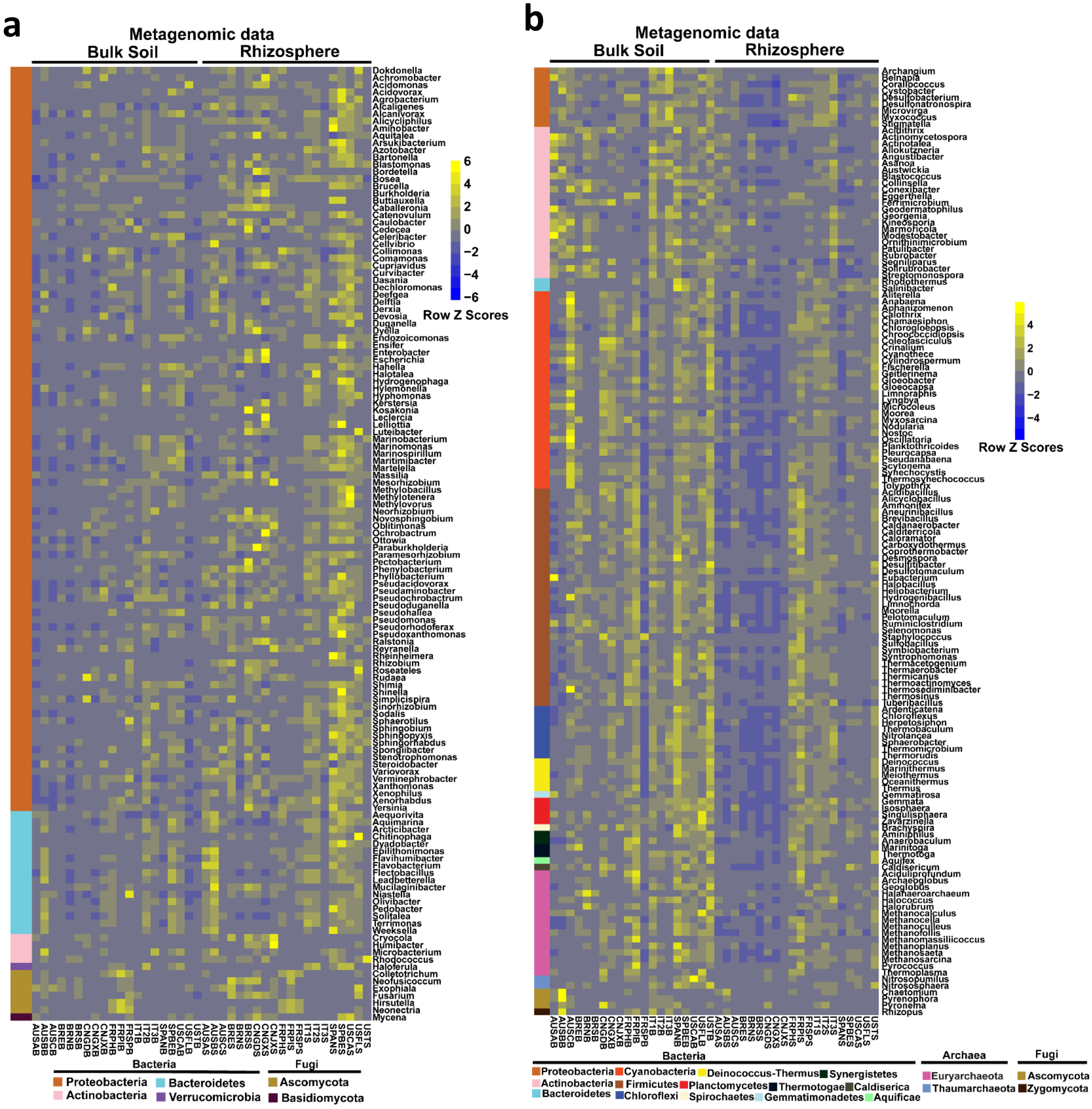

Supplementary Figure 7. The rhizosphere-enriched and rhizosphere-depleted genera using metagenomic data. (a) The relative abundance of rhizosphere-enriched genera. (b) The relative abundance of rhizosphere-depleted genera. Scale, relative abundance of genus at row normalization by removing the mean (centering) and dividing by the standard deviation (scaling). The color from blue to yellow represents a relative abundance of each taxa from low to high.

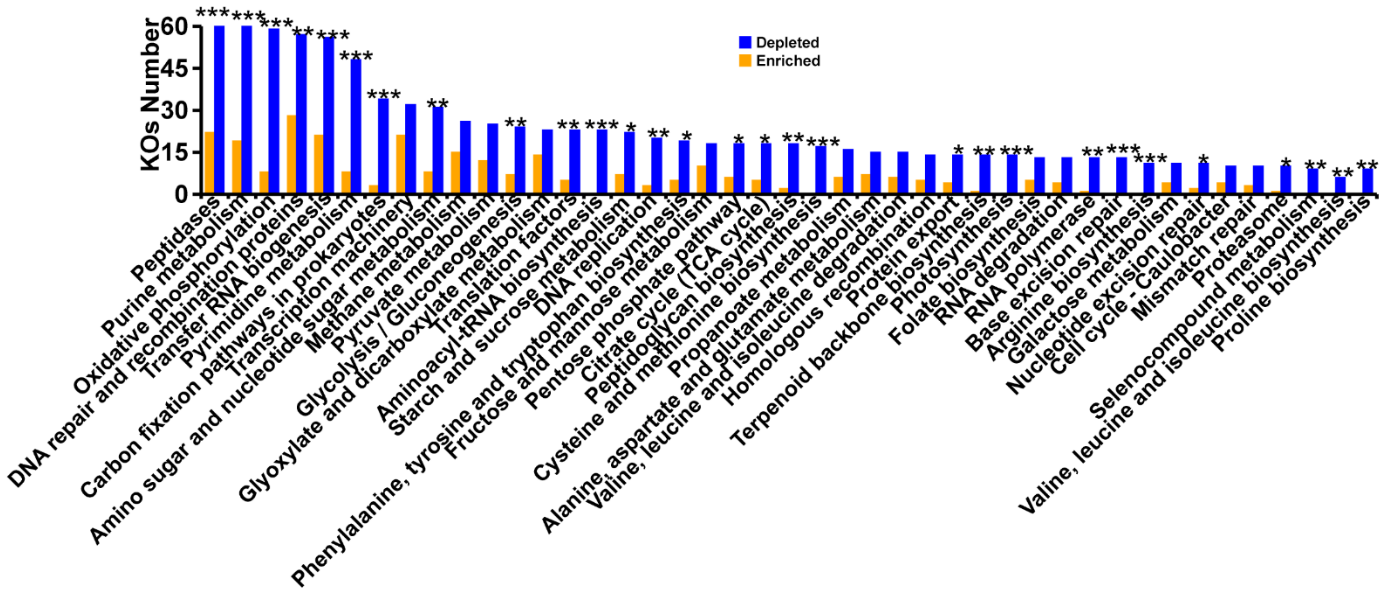

Supplementary Figure 8. The distribution of universally depleted KOs for rhizosphere microbiome on KEGG level 3 pathway. *, adjust P-value <0.05; **, adjust P-value <0.01; ***, adjust P-value <0.001; Fisher's exact test and adjust by BH method.

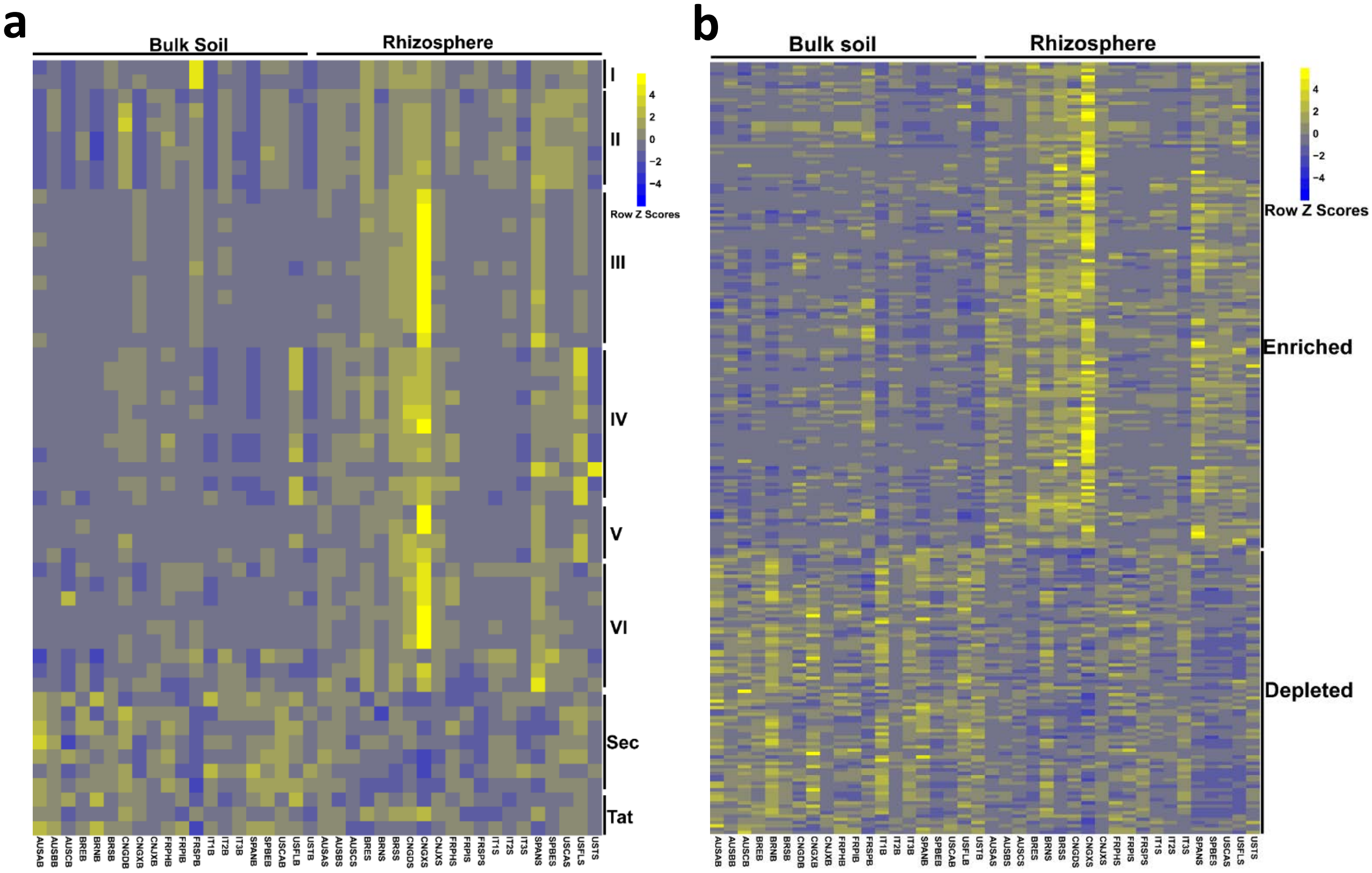

Supplementary Figure 9. The relative abundance of core and universally depleted KOs for rhizosphere microbiome involved in bacterial secretion system, including type I, II, III, IV, V, VI, Sec and Tat (a) and two component system (b). Scale, relative abundance of KO at row normalization by removing the mean (centering) and dividing by the standard deviation (scaling). The color from blue to yellow represents a relative abundance of each KO from low to high.

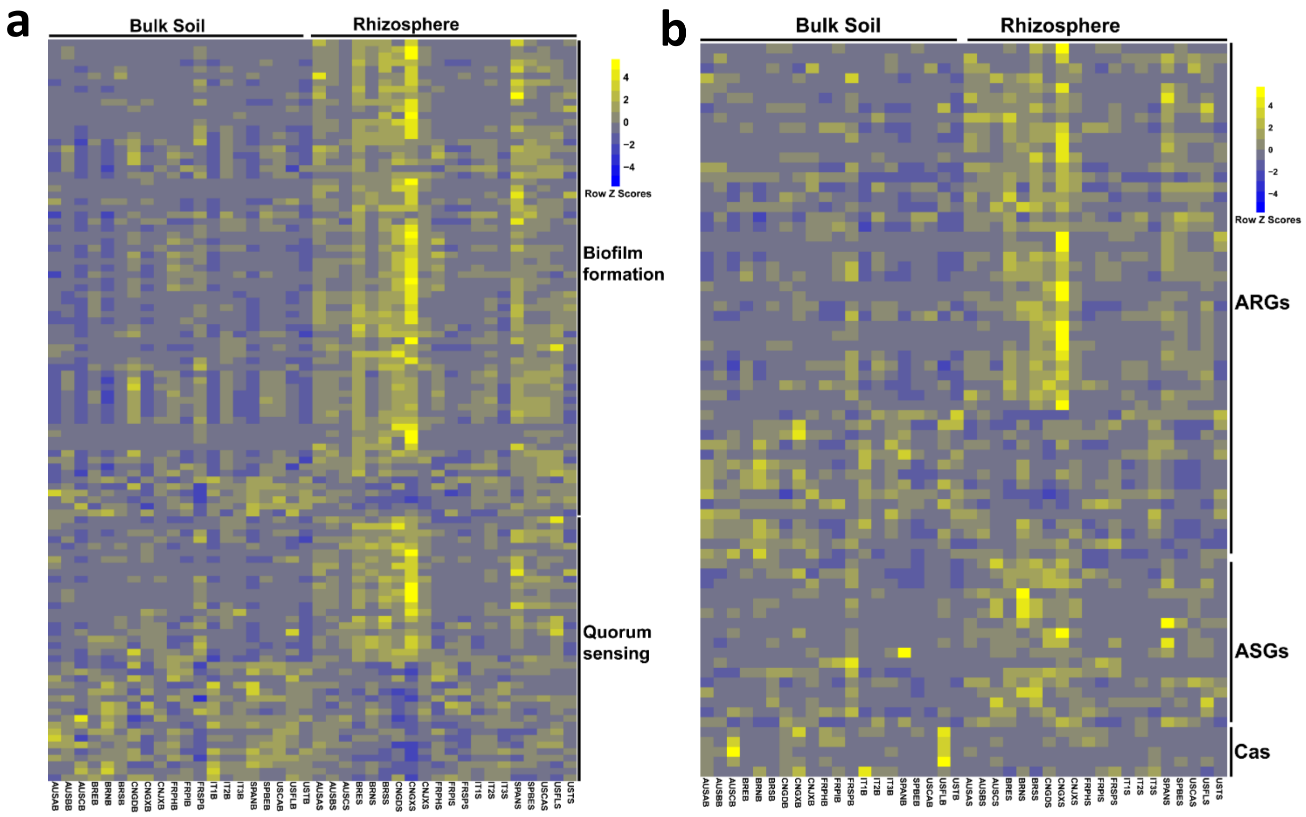

Supplementary Figure 10. The relative abundance of core and universally depleted KOs for rhizosphere microbiome involved in biofilm formation and quorum sensing (a) and antimicrobial resistant genes (ARGs), antibiotic synthesis genes (ASGs) and CRISPR associated proteins (CAS) (b). Scale, relative abundance of KO at row normalization by removing the mean (centering) and dividing by the standard deviation (scaling). The color from blue to yellow represents a relative abundance of each KO from low to high.

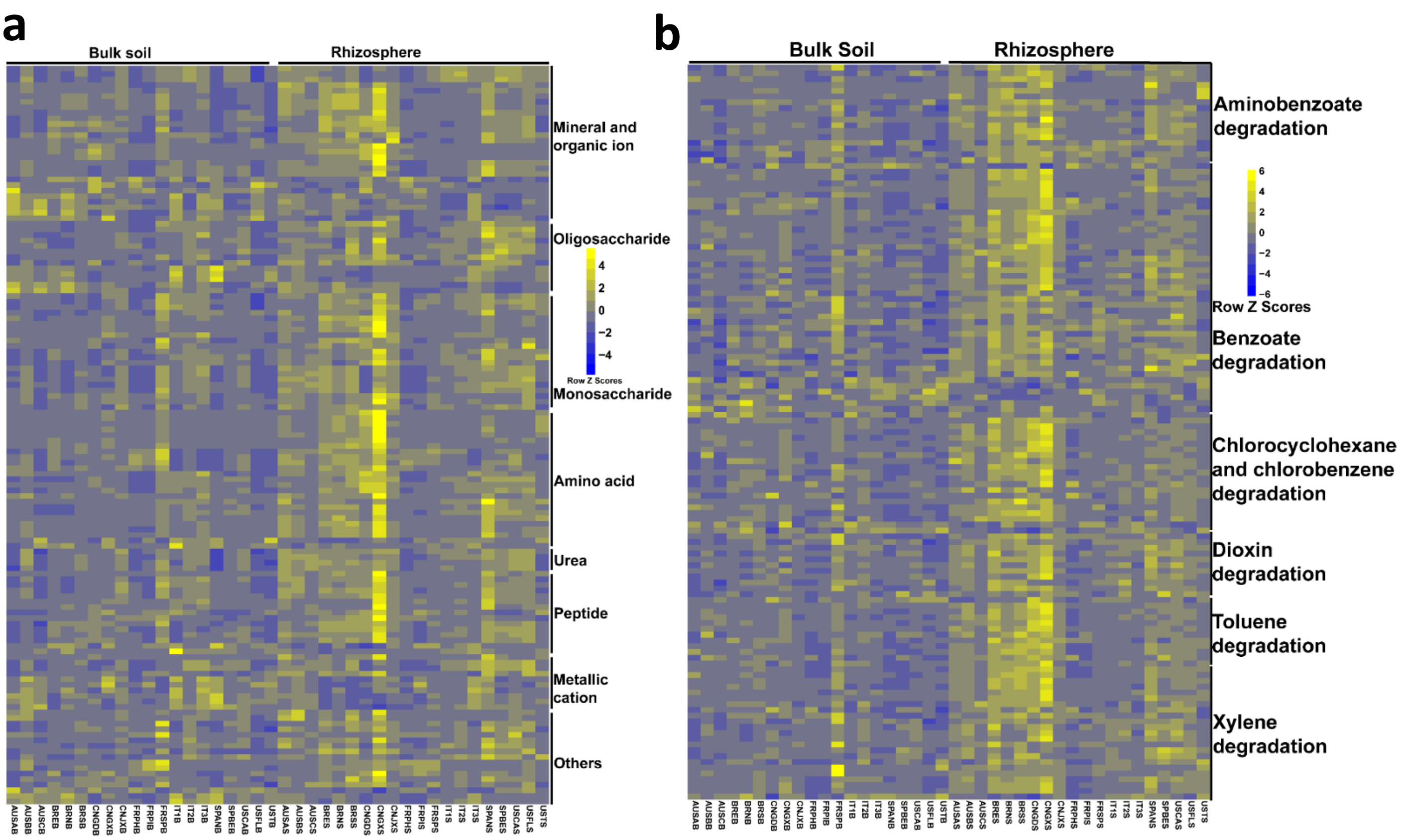

Supplementary Figure 11. The relative abundance of core and universally depleted KOs for rhizosphere microbiome involved in ABC transporters (a) and xenobiotics biodegradation and metabolism (b). Scale, relative abundance of KO at row normalization by removing the mean (centering) and dividing by the standard deviation (scaling). The color from blue to yellow represents a relative abundance of each KO from low to high.

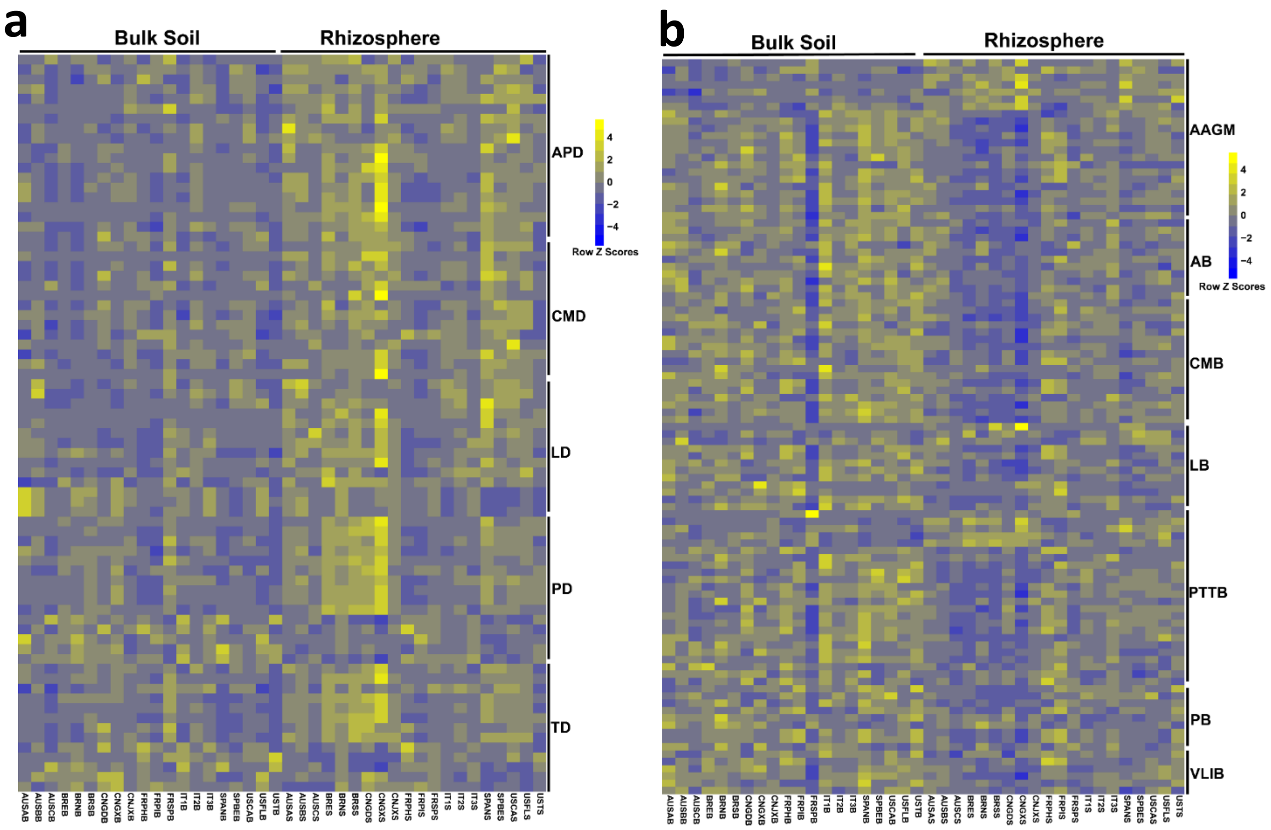

Supplementary Figure 12. The relative abundance of core and universally depleted KOs for rhizosphere microbiome involved in amino acid degradation (a) and biosynthesis (b), respectively. APD, arginine and proline degradation; CMD, cysteine and methionine degradation; LD, lysine degradation; PD, phenylalanine degradation; TD, tyrosine degradation. AAGM, alanine , aspartate and glutamate metabolism; AB, Arginine biosynthesis; CMB, cysteine and methionine biosynthesis; LB, lysine biosynthesis; PTTB, phenylalanine, tyrosine and tryptophan biosynthesis; PB, Proline biosynthesis; VLIB, Valine, leucine and isoleucine biosynthesis. Scale, relative abundance of KO at row normalization by removing the mean (centering) and dividing by the standard deviation (scaling). The color from blue to yellow represents a relative abundance of each KO from low to high.

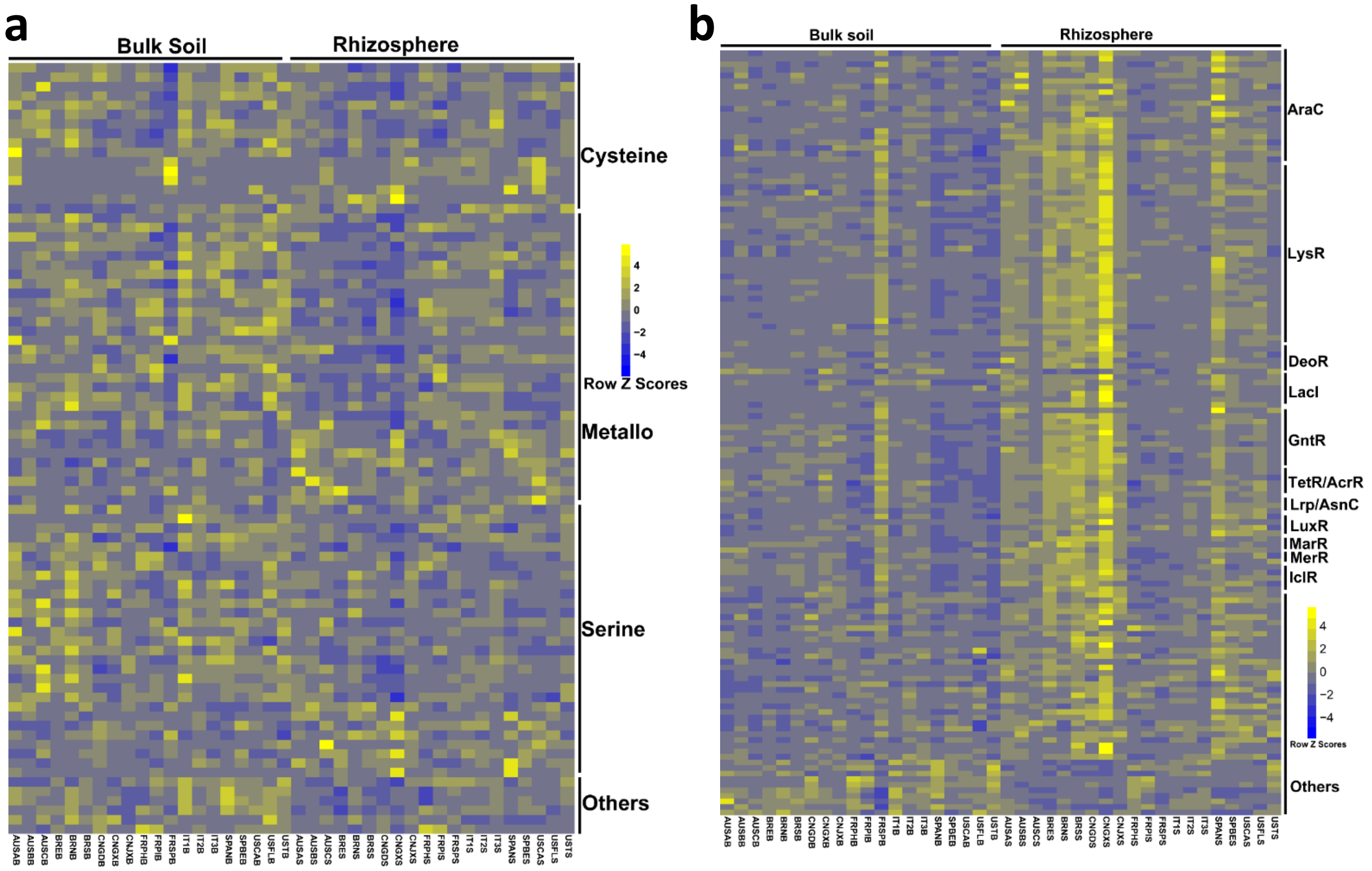

Supplementary Figure 13. The relative abundance of core and universally depleted KOs for rhizosphere microbiome involved in peptidases (a) and transcription factors (b). Scale, relative abundance of KO at row normalization by removing the mean (centering) and dividing by the standard deviation (scaling). The color from blue to yellow represents a relative abundance of each KO from low to high.

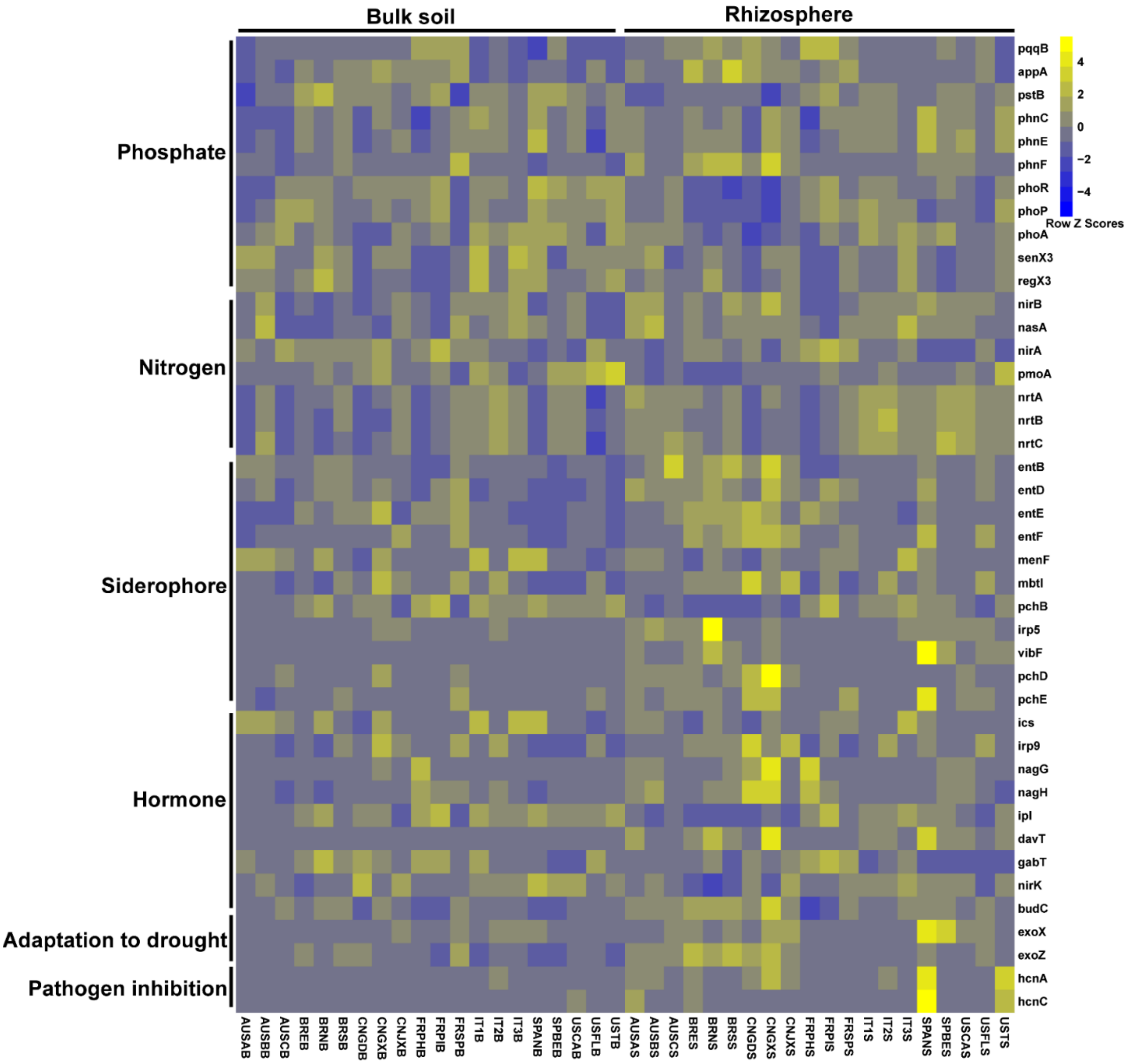

Supplementary Figure 14. The relative abundance of core and universally depleted KOs for rhizosphere microbiome involved in plant nutrition promotion, plant hormone balance, adaptation to drought and pathogen inhibition. Scale, relative abundance of KO at row normalization by removing the mean (centering) and dividing by the standard deviation (scaling). The color from blue to yellow represents a relative abundance of each KO from low to high.

Supplementary Tables

Supplementary Table 1. Summary of the rhizosphere soil and the corresponding bulk soil samples collected from citrus groves worldwide.

CODE	LOCATION	LONGITUDE	LATITUDE	ROOTSTOCK	SCION	AGE	COUNTRY	CONTINENT
UST	Weslaco, Texas	97.96 W	26.17 N	Sour orange	Valencia sweet orange	10	United States	North America
USCA	Riverside, California	117.42 W	33.89 N	Carrizo	Washington Navel	23	United States	North America
USFL	Lake Alfred, Florida	81.71 W	28.11 N	Swingle	Hamlin sweet orange	18	United States	North America
BRS	South Sao Paulo	48.32 W	23.61 S	Rangpur lime	Pera sweet orange	NA	Brazil	South America
BRE	East Sao Paulo	47.03 W	22.38 S	Rangpur lime	Pera sweet orange	NA	Brazil	South America
BRN	North Sao Paulo	49.25 W	20.52 S	Rangpur lime	Pera sweet orange	NA	Brazil	South America
CNGX	Guangxi	108.18 E	23.07 N	Poncirus trifoliata	Shatangju mandarin	NA	China	Asia
CNGD	Guangdong	112.82 E	23.64 N	Poncirus trifoliata	Shatangju mandarin	NA	China	Asia
CNJX	Jiangxi	114.52 E	25.46 N	Poncirus trifoliata	Newhall navel orange	NA	China	Asia
IT1	calabria,Bovalino	16.24 E	38.28 N	Troyer citrange	Tarocco	12	Italy	Europe
IT2	Sicily, Scordia	14.88 E	37.28 N	Troyer citrange	Tarocco	10	Italy	Europe
IT3	Sicily, Paterno	14.81 E	37.49 N	Troyer citrange	Tarocco	12	Italy	Europe
SPBE	Betera, Valencia	0.36 W	39.58 N	Carrizo	lane-late	11	Spain	Europe
SPAN	Carmona, Sevilla, Andalucia	5.81 W	37.51 N	Carrizo	Delta Seedless	13	Spain	Europe
AUSC	Mundubbera, Queensland	151.30 E	25.59 S	Swingle	NA	10-15	Australia	Oceania
AUSB	Griifith, NSW	146.22 E	34.17 S	Swingle	NA	10-15	Australia	Oceania
AUSA	Mildura, Victoria	142.15 E	34.18 S	Swingle	NA	10-15	Australia	Oceania
SANE*	Nelspruit	30.99 E	25.44 S	Swingle	Valencia sweet orange	16	South Africa	Africa
SAHO*	Hoedspruit	30.72 E	24.36 S	Swingle	Valencia sweet orange	9	South Africa	Africa
SAMO*	Mooinooi	27.61 E	25.77 S	Swingle	Valencia sweet orange		South Africa	Africa
FRSP	Le Guillaume	55.57 E	21.35 S	tangor ortanique#		15	Réunion, France	Africa
FRPI	Petite ile, Chemin Jessy	55.56 E	21.34 S	tangor ortanique#		20	Réunion, France	Africa
FRPH	Plaine des cafres, Piton Hyacinthe	55.53 E	21.2 S	tangor ortanique#		8	Réunion, France	Africa

Note: NA, the information was not available. *, only rhizosphere soil samples were collected. For other sites, both rhizosphere and bulk soil samples were collected. #, the trees were from seedlings, not from grafting.

Supplementary Table 2. Summary of the 16S reads

Bulk soil	High quality Tag	Clean Tag	OTU	Rhizosphere	High quality Tag	Clean Tag	OTU
AUSAB	29,130	21,494	2126	AUSAS	27,837	21,361	2438
AUSBB	29,751	23,071	2702	AUSBS	28,639	22,642	2508
AUSCB	30,598	23,459	2356	AUSCS	30,171	22,807	2783
BREB	30,041	22,289	2942	BRES	28,835	22,814	2767
BRNB	29,214	23,171	2623	BRNS	30,035	26,362	2533
BRSB	29,552	21,849	3037	BRSS	28,535	23,911	2454
CNGDB	30,234	21,674	2865	CNGDS	29,221	21,747	2417
CNGXB	29,885	22,703	2817	CNGXS	26,753	22,805	1829
CNJXB	29,652	22,421	2950	CNJXS	29,406	22,330	2825
FRPHB	30,333	22,910	2823	FRPHS	30,314	22,517	2599
FRPIB	30,501	23,100	2514	FRPIS	30,055	22,877	2604
FRSPB	31,088	6,699	1361	FRSPS	30,778	15,557	2238
IT1B	29,870	22,243	2570	IT1S	29,938	21,744	2788
IT2B	29,456	22,486	2863	IT2S	29,781	22,542	2749
IT3B	29,133	22,933	2159	IT3S	29,694	22,971	2307
SPANB	30,825	23,754	2330	SPANS	28,096	24,952	1704
SPBEB	30,897	24,331	2940	SPBES	28,755	23,173	2471
USCAB	30,387	22,536	3211	USCAS	29,960	23,098	3184
USFLB	29,986	23,049	2123	USFLS	29,652	22,565	3176
USTB	30,629	22,667	2242	USTS	29,628	23,865	2331
				SAHOS	29,874	24,437	2239
				SAMOS	28,818	22,536	2313
				SANES	30,297	23,129	3256

Supplementary Table 3. Summary of the ITS reads

Bulk soil	High quality Tag	Clean Tag	OTU	Rhizosphere	High quality Tag	Clean Tag	OTU
AUSAB	24152	23748	409	AUSAS	23894	23392	754
AUSBB	22615	22125	499	AUSBS	23394	22964	627
AUSCB	24418	23870	627	AUSCS	24236	23774	592
BREB	22583	21919	932	BRES	23887	23301	992
BRNB	23592	22862	811	BRNS	24861	24616	588
BRSB	20464	19820	936	BRSS	24602	23773	838
CNGDB	23222	21816	722	CNGDS	22440	21662	722
CNGXB	23916	23248	789	CNGXS	22992	22657	550
CNJXB	23560	22178	581	CNJXS	24216	23254	771
FRSPB	6376	5943	329	FRPIS	24167	23782	722
IT2B	23578	23215	635	FRSPS	10487	10113	571
IT3B	23918	23443	516	IT1S	19224	18931	644
USFLB	23384	22678	813	IT2S	21825	21287	699
USTB	24844	24458	416	IT3S	22939	22469	545
				SAHOS	24920	24779	280
				SAMOS	24530	24028	378
				SANES	24371	24165	497
				SPBES	24445	24217	541
				USCAS	23717	23135	606
				USFLS	23826	23195	810
				USTS	24489	24168	521

Supplementary Table 4. Summary of the metagenomic reads

Bulk soil	clean reads	sickle treated paired	sickle treated sington	sickle/clean	overall citrus mapping rate*	Rhizosphere	clean reads	sickle treated paired	sickle treated sington	sickle/clean	overall citrus mapping rate*
AUSAB	232421170	105656155	9900741	95.18%	0.07%	AUSAS	230361260	108967417	5938730	97.18%	0.10%
AUSBB	232697748	106703069	9131998	95.63%	0.03%	AUSBS	230919620	109765837	5443449	97.43%	0.16%
AUSCB	231774200	105749995	9553184	95.37%	0.03%	AUSCS	231469610	110106009	5380639	97.46%	0.08%
BREB	233035348	109912409	6289030	97.03%	0.18%	BRES	232359704	109370344	6498066	96.94%	1.35%
BRNB	232506112	109634384	6299626	97.02%	0.08%	BRNS	231285736	109143621	6197083	97.06%	1.32%
BRSB	232693870	106610059	9130025	95.55%	0.44%	BRSS	232193680	109377795	6415041	96.98%	0.60%
CNGDB	229998952	107091627	7544882	96.40%	0.04%	CNGDS	231718448	105373714	9433423	95.02%	1.99%
CNGXB	231108704	107314424	7877955	96.28%	0.05%	CNGXS	231704742	109518555	6029448	97.14%	0.22%
CNJXB	231308036	107560506	7685753	96.32%	0.05%	CNJXS	232280492	105175655	9780091	94.77%	0.33%
FRPHB	232413558	107814480	8015428	96.23%	0.03%	FRPHS	230572604	107310508	7532205	96.35%	0.10%
FRPIB	232402148	107980913	7840207	96.30%	0.03%	FRPIS	230632756	107844795	7082604	96.59%	0.12%
FRSPB	231798562	107277214	8063319	96.04%	31.94%	FRSPS	230918916	107712126	7269220	96.44%	4.43%
IT1B	218016080	99971308	8597021	95.65%	0.03%	IT1S	229874840	93869918	18181483	89.58%	2.88%
IT2B	231321602	106826550	8421056	96.00%	0.15%	IT2S	232671108	106902513	8791320	95.67%	0.43%
IT3B	232044826	107455369	8011698	96.07%	0.15%	IT3S	267844174	107522376	7725458	83.17%	0.29%
SPANB	231552198	104940445	10255824	95.07%	0.04%	SPANS	230214464	109452600	5412193	97.44%	0.15%
SPBEB	229691642	106490925	7934662	96.18%	0.04%	SPBES	227747236	106371250	7115742	96.54%	0.21%
USCAB	232590868	107541877	8099211	95.96%	0.15%	USCAS	230496564	109674822	5291049	97.46%	0.48%
USFLB	232351870	103905927	11198327	94.26%	0.12%	USFLS	229039886	108922315	5331644	97.44%	0.38%
USTB	209643484	95752797	8413088	95.36%	0.17%	USTS	222093020	104908607	5866617	97.11%	0.10%
						SAHOS	231537168	109603066	5888580	97.22%	0.05%
						SAMOS	229257850	108141439	6167603	97.03%	0.33%
						SANES	231536142	109648157	5835389	97.23%	0.09%

*, the sickle trimmed reads were mapped to the sweet orange, Citrus clementina and Swingle citrumelo genomes using bowtie2 software, and the overall alignment rate was shown here.

Supplementary Table 5. The assembly status of the 9 sample groups (based on contigs ≥200 bp)

group	No. of contigs	total length (bp)	Max (bp)	Average (bp)	N50	Reads utilization rate (%)
Group4	27,664,602	16,035,964,207	289,328	580	599	44
Group9	9,704,470	6,092,582,093	338,689	628	669	46
Group5	21,442,185	12,374,648,103	384,386	577	590	38
Group6	37,146,365	21,562,290,026	278,402	580	608	49
Group7	19,333,433	11,771,617,595	401,613	609	638	48
Group8	29,766,115	18,471,561,284	403,934	621	660	55
Group2	32,593,522	19,131,351,681	674,951	587	625	53
Group1	32,393,177	19,664,889,472	255,355	607	648	54
Group3	13,928,059	8,285,509,004	147,221	595	619	48
Average	24,885,770	14,821,157,052	352,653	598	628	48
