# Supplementary material for: The structure and function of the global citrus rhizosphere microbiome
Source: Nat Commun. 2018 Nov 20;9:4894. doi: 10.1038/s41467-018-07343-2 (PMC6244077; doi:10.1038/s41467-018-07343-2)
Supplement: Supplementary file 3 — Descriptions of Additional files [file 41467_2018_7343_MOESM3_ESM.docx]

**Description of Additional Supplementary Files**

File Name: Supplementary Data 1

Description: The physical and chemical characteristics of soil at each sampling location.

File Name: Supplementary Data 2

Description: The results of comparison analysis across compartments (Bulk soil and rhizosphere) for taxonomic contents at Phylum level using amplicon data. The DESeq2 comparison analysis was performed based on the read count matrix of the phyla across the bulk soil and rhizosphere samples (n=20 for each group in 16S, n=13 for each group in ITS2). P-values for multiple testing were corrected using the BH method in DESeq2. All items with corrected P-values<0.05 were considered significant.

File Name: Supplementary Data 3

Description: The results of comparison analysis across compartments (Bulk soil and rhizosphere) for taxonomic contents at Phylum level using metagenomic data. The DESeq2 comparison analysis was performed based on the read count matrix of the phyla across the bulk soil and rhizosphere samples (n=20 for each group). P-values for multiple testing were corrected using the BH method in DESeq2. All items with corrected P-values<0.05 were considered significant.

File Name: Supplementary Data 4

Description: The results of comparison analysis across compartments (Bulk soil and rhizosphere) for taxonomic contents at genus level using amplicon data. The DESeq2 comparison analysis was performed based on the read count matrix of the genera across the bulk soil and rhizosphere samples (n=20 for each group in 16S, n=13 for each group in ITS2). P-values for multiple testing were corrected using the BH method in DESeq2. All items with corrected P-values<0.05 were considered significant.

File Name: Supplementary Data 5

Description: The results of comparison analysis across compartments (Bulk soil and rhizosphere) for taxonomic contents at genus level using metagenomic data. The DESeq2 comparison analysis was performed based on the read count matrix of the genera across the bulk soil and rhizosphere samples (n=20 for each group). P-values for multiple testing were corrected using the BH method in DESeq2. All items with corrected P-values<0.05 were considered significant.

File Name: Supplementary Data 6

Description: The results of comparison analysis across compartments (Bulk soil and rhizosphere) for functional contents (KOs) using metagenomic data. The DESeq2 comparison analysis was performed based on the read count matrix of the KOs across the bulk soil and rhizosphere samples (n=20 for each group). P-values for multiple testing were corrected using the BH method in DESeq2. All items with corrected P-values<0.05 were considered significant.
