# Supplementary material for: The structure and function of the global citrus rhizosphere microbiome
Source: Nat Commun. 2018 Nov 20;9:4894. doi: 10.1038/s41467-018-07343-2 (PMC6244077; doi:10.1038/s41467-018-07343-2)
Supplement: Supplementary file 10 — Reporting Summary [file 41467_2018_7343_MOESM10_ESM.pdf]

nature research

Corresponding author(s): Nian Wang

Reporting Summary

Nature Research wishes to improve the reproducibility of the work that we publish. This form provides structure for consistency and transparency in reporting. For further information on Nature Research policies, see Authors & Referees and the Editorial Policy Checklist.

Statistical parameters

When statistical analyses are reported, confirm that the following items are present in the relevant location (e.g. figure legend, table legend, main text, or Methods section).

n/a	Confirmed	
☐	☒	The exact sample size (n) for each experimental group/condition, given as a discrete number and unit of measurement
☒	☐	An indication of whether measurements were taken from distinct samples or whether the same sample was measured repeatedly
☐	☒	The statistical test(s) used AND whether they are one- or two-sided<br>*Only common tests should be described solely by name; describe more complex techniques in the Methods section.*
☒	☐	A description of all covariates tested
☐	☒	A description of any assumptions or corrections, such as tests of normality and adjustment for multiple comparisons
☐	☒	A full description of the statistics including central tendency (e.g. means) or other basic estimates (e.g. regression coefficient) AND variation (e.g. standard deviation) or associated estimates of uncertainty (e.g. confidence intervals)
☐	☒	For null hypothesis testing, the test statistic (e.g. F, t, r) with confidence intervals, effect sizes, degrees of freedom and P value noted<br>*Give P values as exact values whenever suitable.*
☒	☐	For Bayesian analysis, information on the choice of priors and Markov chain Monte Carlo settings
☒	☐	For hierarchical and complex designs, identification of the appropriate level for tests and full reporting of outcomes
☒	☐	Estimates of effect sizes (e.g. Cohen's d, Pearson's r), indicating how they were calculated
☒	☐	Clearly defined error bars<br>*State explicitly what error bars represent (e.g. SD, SE, CI)*

Our web collection on statistics for biologists may be useful.

Software and code

Policy information about availability of computer code

Data collection	All published open source softwares were described in method section. And custom bioinformatic code is freely accessible through our website (https://db.cngb.org/icrm).
Data analysis	All published open source softwares were described in method section. And custom bioinformatic code is freely accessible through our website (https://db.cngb.org/icrm).

For manuscripts utilizing custom algorithms or software that are central to the research but not yet described in published literature, software must be made available to editors/reviewers upon request. We strongly encourage code deposition in a community repository (e.g. GitHub). See the Nature Research guidelines for submitting code & software for further information.

Data

Policy information about availability of data

All manuscripts must include a data availability statement. This statement should provide the following information, where applicable:

- Accession codes, unique identifiers, or web links for publicly available datasets
- A list of figures that have associated raw data
- A description of any restrictions on data availability

The raw sequencing reads were deposited in the NCBI Bioproject database under the accession number PRJNA362455. The nonredundant reference catalog of the

citrus rhizosphere and bulk soil microbiome is freely accessible through our website (https://db.cngb.org/icrm). Other data supporting the findings of the study are available in this article and its Supplementary Information files, or from the corresponding authors upon request.

Field-specific reporting

Please select the best fit for your research. If you are not sure, read the appropriate sections before making your selection.

☒ Life sciences ☐ Behavioural & social sciences ☐ Ecological, evolutionary & environmental sciences

For a reference copy of the document with all sections, see nature.com/authors/policies/ReportingSummary-flat.pdf

Life sciences study design

All studies must disclose on these points even when the disclosure is negative.

Sample size	To represent the biogeographical differences in rhizosphere communities, we collected samples from 23 locations in eight citrus producing countries spanning all six citrus-producing continents. In total, we obtained 23 rhizosphere soil samples and 20 bulk soil samples from 23 representative locations in the USA, China, Brazil, Spain, Italy, Australia, France and South Africa, (no bulk soil samples were collected from the locations in South Africa)
Data exclusions	For the paired DESeq2 comparison analysis of the genera and KOs across the bulk soil and rhizosphere samples, the samples from South Africa were excluded due to the absence of bulk soil samples in this location
Replication	To represent the biogeographical differences in rhizosphere communities, we collected samples from 23 locations in eight citrus producing countries spanning all six citrus-producing continents. In total, we obtained 23 rhizosphere soil samples and 20 bulk soil samples from 23 representative locations in the USA, China, Brazil, Spain, Italy, Australia, France and South Africa, (no bulk soil samples were collected from the locations in South Africa)
Randomization	Not applicable
Blinding	Not applicable

Reporting for specific materials, systems and methods

Materials & experimental systems

n/a	Involved in the study	
☒	☐	Unique biological materials
☒	☐	Antibodies
☒	☐	Eukaryotic cell lines
☒	☐	Palaeontology
☒	☐	Animals and other organisms
☒	☐	Human research participants

Methods

n/a	Involved in the study	
☒	☐	ChIP-seq
☒	☐	Flow cytometry
☒	☐	MRI-based neuroimaging
